# Splenic cDC1 efferocytosis and cross-presentation to CD8 T cells are promoted by GPR34 and lysophosphatidylserine

**DOI:** 10.1084/jem.20250887

**Published:** 2025-11-10

**Authors:** Hanson Tam, Haolin Shen, Ying Xu, Jinping An, Jason G. Cyster

**Affiliations:** 1 Howard Hughes Medical Institute, University of California, San Francisco, San Francisco, CA, USA; 2 Department of Microbiology and Immunology, University of California, San Francisco, San Francisco, CA, USA; 3 Medical Scientist Training Program, University of California, San Francisco, San Francisco, CA, USA; 4 Biomedical Sciences Graduate Program, University of California, San Francisco, San Francisco, CA, USA

**Keywords:** spleen, conventional dendritic cells type 1, efferocytosis, cross-presentation, GPR34, PLA1A

## Abstract

Spleen conventional dendritic cells type 1 (cDC1) take up apoptotic cells (AC) and cross-present associated antigens to CD8 T cells. The receptors promoting AC uptake are incompletely defined. Here we tested the function of GPR34, a receptor that responds to the phosphatidylserine (PS) catabolite lysoPS. GPR34 deficiency led to reduced AC uptake by cDC1 but not cDC2 or macrophages. Uptake of soluble antigen or heat-killed bacteria was unaffected whereas uptake of eryptotic RBCs was reduced. Using AC harboring OVA, activation and proliferation of OT-I T cells were compromised in GPR34-deficient mice. Reciprocally, GPR34 overexpression led to enhanced AC uptake and OT-I proliferation. The enzymes PLA1A and ABHD16A have been implicated in generating lysoPS that can act on GPR34. PLA1A but not ABHD16A deficiency was associated with a reduced OT-I response to AC-associated OVA. In conclusion, we identify a receptor requirement for cDC1 efferocytosis and cross-presentation and suggest a model where PLA1A catabolizes PS on AC to generate GPR34 ligands.

## Summary

Tam et al. identify GPR34 as a splenic cDC1 receptor that is required for efferocytosis and for the cross-presentation of apoptotic cell-associated antigen to CD8 T cells. The lysophosphatidylserine-generating enzyme PLA1A acts in the GPR34 pathway to support this response.

## Introduction

Apoptotic cells (AC) that enter circulation are efficiently captured and cleared in the spleen. While many AC arriving in the spleen are engulfed by macrophages, some are taken up by conventional dendritic cells type 1 (cDC1) (Miyake et al., 2007; Morelli et al., 2003; Qiu et al., 2009). At homeostasis, cDC1 are present in the splenic red pulp and marginal zone (MZ), sites of cell arrival in the spleen via the blood (Alexandre et al., 2016; Calabro et al., 2016; Dorner et al., 2009; Qiu et al., 2009). Using AC loaded with model antigens such as OVA, cDC1 have been shown to cross-present AC-associated antigens to CD8 T cells (Liu et al., 2002; Moore et al., 1988). In the absence of other stimuli, the CD8 T cells become activated and divide but have a diminished ability to respond to a subsequent antigen exposure (Bosteels et al., 2023; Liu et al., 2002; Steinman et al., 2000). While the cDC1 changes following AC engulfment and the properties of the associated CD8 T cell response have been extensively studied, there has been limited investigation of the AC encounter and uptake mechanisms used by cDC1.

The best defined “eat-me” signal displayed by AC is phosphatidylserine (PS), a membrane lipid that is abundant in the inner leaflet of healthy cells and is flipped to the outer leaflet as an early event during apoptosis (Arandjelovic and Ravichandran, 2015). Multiple PS receptors have been identified including TIM-3 (HAVCR2), TIM-4, BAI1, TREM2, Stabilin-1, Stabilin-2, CD300, MFGE8, and MERTK family members that bind PS via GAS6 or PROS1 (Arandjelovic and Ravichandran, 2015; Freeman et al., 2010). Among these receptors, TIM-3 and CD300 are expressed by cDC1 (https://www.immgen.org/). As well as receptors for PS, receptors that respond to metabolites released from AC have been suggested to help phagocytes find AC (Arandjelovic and Ravichandran, 2015). However, the receptors acting on cDC1 to favor AC encounter or uptake are poorly understood (Belz et al., 2002; Nakayama et al., 2009; Perry et al., 2018; Schulz et al., 2002).

GPR34 is a lysoPS receptor that can couple to G_αi_-containing heterotrimeric G proteins (Inoue et al., 2019; Izume et al., 2024). The receptor can respond to both *sn*-1 and *sn*-2 forms of lysoPS, though in vitro studies have found *sn*-2 lysoPS to be markedly more potent than its *sn*-1 counterpart (Kitamura et al., 2012; Uwamizu et al., 2015). The *sn*-2 form of lysoPS can be generated from PS by the extracellularly secreted enzyme PLA1A (also known as PS-PLA1), while *sn*-1 lysoPS can be generated from PS by ABHD16A and by some PLA2 enzymes (Hosono et al., 2001; Kamat et al., 2015; Makide and Aoki, 2013). The function of GPR34 in vivo remains poorly understood, but roles in macrophages and innate lymphoid cells (ILC) have been reported (Lin et al., 2024; Lou et al., 2022; Preissler et al., 2015; Wang et al., 2021; Yan et al., 2024). Despite PS exposure being a cardinal feature of apoptosis, there have so far been limited data connecting apoptosis with GPR34 function. In a mouse colitis study, apoptotic neutrophils were a source of GPR34^+^ ILC3-engaging lysoPS, though the enzyme involved in lysoPS generation was not defined (Wang et al., 2021).

GPR34 gain-of-function (GOF) alleles have been identified in mucosa-associated lymphoid tissue B cell lymphoma (Ansell et al., 2012; Baens et al., 2012). Expression of a GPR34 GOF variant in mouse B lineage cells promoted plasma cell and memory B cell accumulation in the peritoneal cavity in a PLA1A-dependent manner (Tam et al., 2024). The source of PS in this setting was unclear. In another study, ABHD16A was suggested to generate GPR34-engaging lysoPS in the tumor microenvironment though it was unclear if AC were needed (Yan et al., 2024). Thus, the circumstances under which PS exposed on AC is associated with lysoPS generation and the enzyme(s) involved in GPR34 ligand production remain poorly understood.

Here we explored the hypothesis that GPR34 expression in cDC1 contributes to uptake of AC in a manner dependent on an *sn*-2 lysoPS-generating enzyme. We found that splenic cDC1 uptake of AC and cross-presentation of antigen to CD8 T cells were promoted by cDC1-intrinsic GPR34 expression. The extracellular *sn*-2 lysoPS-generating PLA1A enzyme was required for GPR34-mediated AC antigen cross-presentation by cDC1.

## Results and Discussion

### GPR34 promotes splenic cDC1 efferocytosis

**Figure 1.**
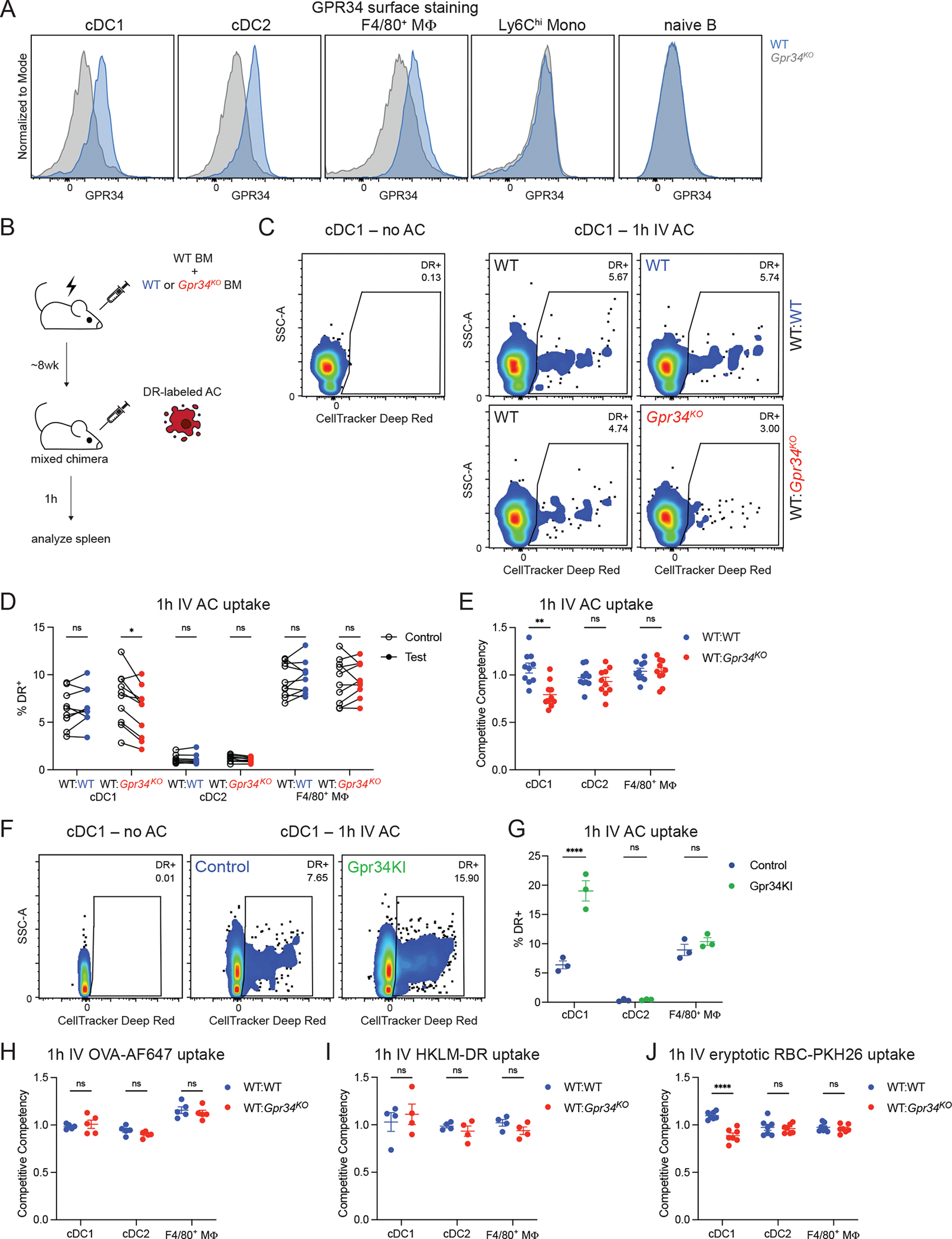
GPR34 promotes splenic cDC1 efferocytosis. **(A-J)** Splenic immune cells were analyzed by flow cytometry. **(A)** Representative flow cytometry histograms of polyclonal anti-GPR34 antibody surface staining of the indicated populations from WT (blue) and *Gpr34*^*KO*^ (gray) mice. **(B-E)** cDC1, cDC2, and F4/80^+^ macrophage (MΦ) uptake of DR-labeled AC was analyzed at 1 h after IV transfer to mixed chimeric mice that had been made by reconstituting WT hosts with a 50:50 mixture of control WT BM and of test WT or *Gpr34*^*KO*^ BM. **(B)** Schematic of the generation of mixed chimeric mice and subsequent in vivo AC uptake assay. **(C)** Representative flow cytometry plots of cDC1 from mice unexposed to DR-labeled AC or from the indicated control and test compartments of mixed chimeric mice gated for DR^+^ cells and annotated with DR^+^ frequency. **(D)** Frequencies of DR^+^ cells within the indicated populations (WT:WT, *n* = 10; WT:*Gpr34*^*KO*^, *n* = 10). Lines connect control and test data from individual mice. **(E)** Competitive competencies of DR^+^ frequency for the indicated populations. Competitive competency was calculated as the ratio of values between the test and control compartments within each mixed chimeric mouse. Data are from the same mice as in D. **(F and G)** cDC1, cDC2, and F4/80^+^ MΦ uptake of DR-labeled AC was analyzed at 1 h after IV transfer to control (*Xcr1*^*Cre*^) and Gpr34KI (*Xcr1*^*Cre*^*R26*^*LSL-Gpr34Q340X-IRES-GFP*^) mice. **(F)** Representative flow cytometry plots of cDC1 from mice unexposed to DR-labeled AC or from control and Gpr34KI mice gated for DR^+^ cells and annotated with DR^+^ frequency. **(G)** Frequencies of DR^+^ cells within the indicated populations (control, *n* = 3; Gpr34KI, *n* = 3). **(H)** Competitive competencies of AF647^+^ frequency for the indicated populations in mixed chimeric mice 1 h after IV injection of soluble OVA-AF647 (WT:WT, *n* = 5; WT:*Gpr34*^*KO*^, *n* = 5). **(I)** Competitive competencies of DR^+^ frequency for the indicated populations in mixed chimeric mice 1 h after IV transfer of DR-labeled HKLM (WT:WT, *n* = 4; WT:*Gpr34*^*KO*^, *n* = 4). **(J)** Competitive competencies of PKH26^+^ frequency for the indicated populations in mixed chimeric mice 1 h after IV transfer of PKH26-labeled eryptotic RBCs (WT:WT, *n* = 7; WT:*Gpr34*^*KO*^, *n* = 7). In E and G-J, each data point represents an individual mouse, lines indicate means, and error bars represent SEM. Data were pooled from two or more independent experiments, except in G, which was from one experiment representative of two. Statistical significance was determined by a paired *t* test (D) or unpaired *t* test (E and G-J) corrected for multiple comparisons (Holm-Šídák). ns, not significant; *P < 0.05; **P < 0.01; ****P < 0.0001.

Flow cytometry analysis of mouse splenic myeloid cells identified GPR34 expression on cDC1, cDC2, and F4/80^+^ macrophages and a lack of expression by Ly6C^hi^ monocytes and naive B cells (Fig. 1 A and Fig. S1 A) in accord with transcript data for these cell types (https://www.immgen.org/; Fig. S1 B; Jager et al., 2016). To test for a possible role of GPR34 in cDC1 efferocytosis, we used a Deep Red (DR) dye-labeled apoptotic thymocyte and splenocyte intravenous (IV) transfer approach similar to other studies of spleen myeloid cell efferocytosis (Bosteels et al., 2023; den Haan et al., 2000; Qiu et al., 2009). In preliminary experiments, we found that the extent of AC uptake, as measured by the percent of DR^+^ cDC, varied between animals despite careful efforts to transfer equivalent amounts of apoptotic material (unpublished data). To allow measurements to be made even with mouse-to-mouse variability, we used a mixed bone marrow (BM) chimeric approach that enables comparison of AC uptake by control and GPR34 knockout cells within the same animal (Fig. 1 B). This approach revealed a deficiency in efferocytosis in GPR34 null cDC1 1 h after AC IV transfer (Fig. 1, C and D). In agreement with previous findings, cDC1 captured more AC than cDC2. F4/80^+^ red pulp macrophages were the most efficient at efferocytosis but were unaffected by GPR34 deficiency (Fig. 1 D). To permit comparison of the impact of GPR34 deficiency on AC uptake across the different cell populations, we plotted competitive competency, the ratio of values between the test and control compartments within each mixed chimeric mouse, confirming the efferocytosis defect in cDC1 (Fig. 1 E). The frequency of splenic cDC and proportion of cDC1 and cDC2 were unaffected by GPR34 deficiency (Fig. S1, C and D) and XCR1^+^ cDC1 were distributed similarly in WT and GPR34-deficient spleens (Fig. S1 E).

We next asked whether GPR34 overexpression was sufficient to improve efferocytosis. Mice harboring a GOF *Gpr34 Q340X* variant in the *Rosa26* locus with an upstream LoxP-Stop-LoxP cassette, referred to as GPR34 knock-in (KI) (Tam et al., 2024), were crossed with the cDC1-specific *Xcr1*^*Cre*^ driver line (Ferris et al., 2020). 1 h after AC injection, GPR34 KI mice showed several fold elevated AC uptake compared with matched control mice (Fig. 1, F and G). cDC2 and F4/80^+^ macrophages that lack GPR34 KI expression did not show any change in AC uptake (Fig. 1 G).

In contrast to AC uptake, splenic cDC1 internalization of soluble OVA was not affected by GPR34 deficiency (Fig. 1 H). Uptake of dye-labeled heat-killed *Listeria monocytogenes* (HLKM) was also not impacted by GPR34 deficiency (Fig. 1 I). In contrast, RBCs that had been ionomycin-treated to undergo eryptosis and exposure of surface PS (Larsson et al., 2016) showed a similar cDC1 dependence on GPR34 for uptake as AC, while cDC2 and macrophage uptake of eryptotic RBCs was unaffected by GPR34 deficiency (Fig. 1 J).

Using in vitro transwell assays, splenic cDC1 showed a migratory response to the CCR7 ligand CCL19, but we were unable to detect migration to lysoPS (Fig. S1 F). This may be a consequence of the rapid changes that occur in cDC upon isolation and culture (Steinman et al., 2003). However, confirming the promigratory activity of GPR34 (Kitamura et al., 2012; Tam et al., 2024), cDC1 from GPR34 KI mice underwent migration in response to lysoPS (Fig. S1 F). The reduced CCL19 response of GPR34 KI cells may reflect competition between the over-expressed GPR34 GOF allele and CCR7 for G_i_ coupling. We also attempted in vitro phagocytosis assays using DR-labeled AC and incubation with total splenocytes or enriched cDC. However, the extent of AC uptake under in vitro conditions was highly variable and we were unable to measure the contribution of GPR34 to cDC1 efferocytosis in the in vitro setting (unpublished data).

### CD8 T cell activation in response to AC-derived antigen is promoted by GPR34

**Figure 2.**
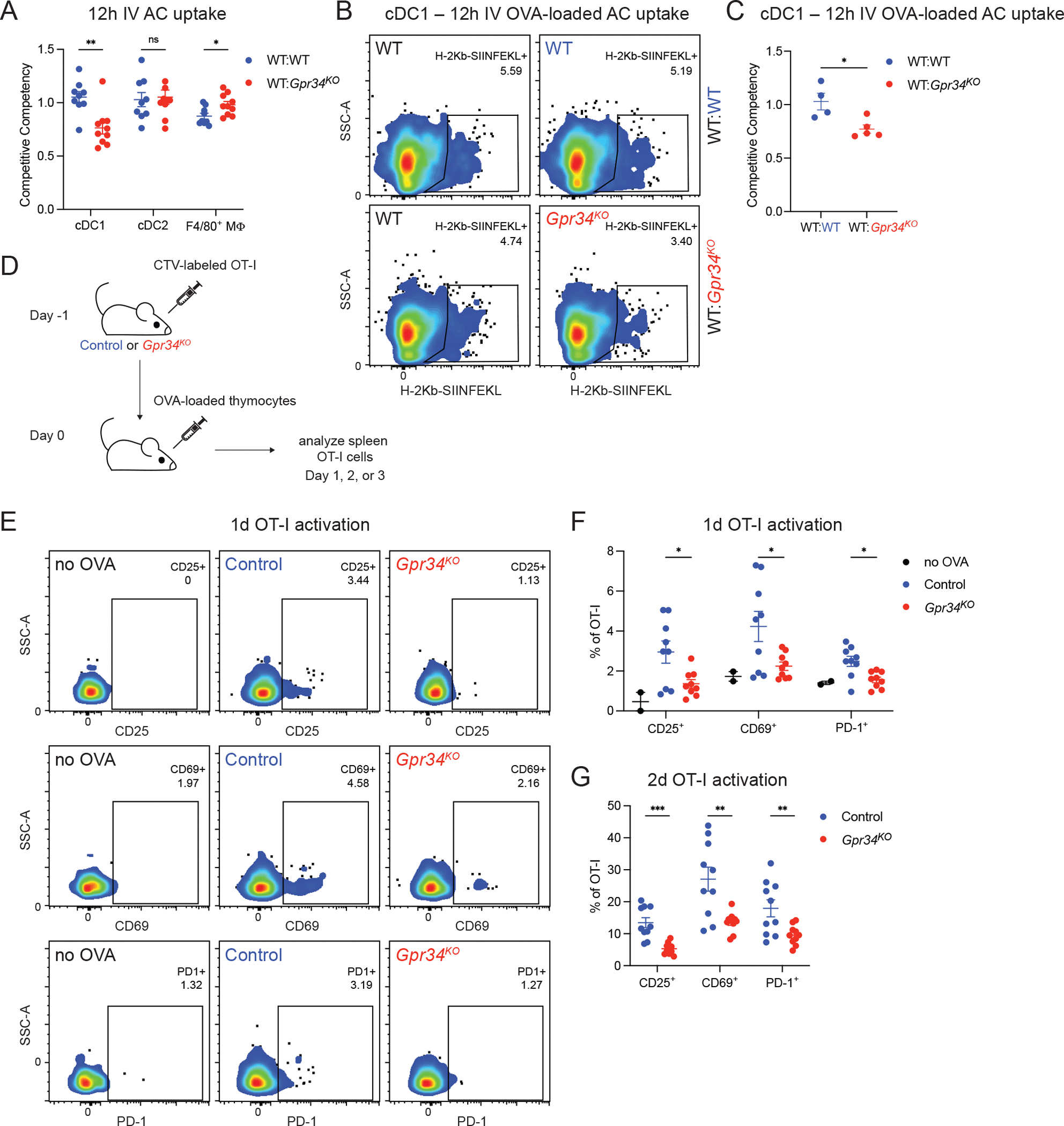
Activation of AC-associated antigen-specific CD8 T cells depends on GPR34. **(A-G)** Splenic immune cells were analyzed by flow cytometry. **(A)** Competitive competencies of DR^+^ frequency for the indicated populations in mixed chimeric mice 12 h after IV transfer of DR-labeled AC (WT:WT, *n* = 9; WT:*Gpr34*^*KO*^, *n* = 10). **(B)** Representative flow cytometry plots of cDC1 from the indicated control and test compartments of mixed chimeric mice 12 h after IV injection of OVA-loaded AC gated for and annotated with H-2K^b^-SIINFEKL^+^ frequency. **(C)** Competitive competencies of H-2K^b^- SIINFEKL^+^ frequency for the indicated cDC1 populations in mixed chimeric mice 12 h after IV injection of OVA-loaded AC (WT:WT, *n* = 4; WT:*Gpr34*^*KO*^, *n* = 5). **(D-G)** Naive CD8 T cells were isolated from OT-I mice, labeled with CTV, and IV transferred to control (WT or *Gpr34*^*Het*^) and *Gpr34*^*KO*^ mice the day before IV transfer of OVA-loaded thymocytes. OT-I cells were analyzed at 1 or 2 days (and in Fig. 3, at 3 days). **(D)** Schematic of in vivo AC-associated antigen cross-presentation assay. **(E and F)** Analysis of OT-I response in mice unexposed to OVA or in control and *Gpr34*^*KO*^ mice 1 day after transfer of OVA-loaded thymocytes. **(E)** Representative flow cytometry plots of OT-I cells, gated for CD25^+^, CD69^+^, and PD-1^+^ cells and annotated with the respective frequencies. **(F)** Frequencies of CD25^+^, CD69^+^, and PD-1^+^ OT-I cells (no OVA, *n* = 2; control, *n* = 9; *Gpr34*^*KO*^, *n* = 9). **(G)** Frequencies of CD25^+^, CD69^+^, and PD-1^+^ OT-I cells 2 days after transfer of OVA-loaded thymocytes (control, *n* = 10; *Gpr34*^*KO*^, *n* = 10). In A, C, F, and G, each data point represents an individual mouse, lines indicate means, and error bars represent the SEM. Data were pooled from two or more independent experiments. Statistical significance was determined by an unpaired *t* test corrected for multiple comparisons (Holm-Šídák). ns, not significant; *P < 0.05; **P < 0.01; ***P < 0.001.

The reduced AC uptake by GPR34-deficient cDC1 was still evident 12 h after AC transfer (Fig. 2 A). AC uptake by cDC1 promotes increased CCR7 and CD86 expression (Bosteels et al., 2023). CCR7 upregulation occurred on both WT and GPR34-deficient cDC1 that had taken up DR-labeled AC, though the extent of upregulation was slightly less for GPR34-deficient cells (Fig. S2 A). Lower CCR7 expression by GPR34-deficient cDC1 may reflect less uptake of AC. CD86 was also upregulated on WT and GPR34-deficient cDC1 after AC uptake, with a trend for lower upregulation in the GPR34-deficient cells (Fig. S2 B).

To test the impact of reduced efferocytosis on antigen presentation and CD8 T cell activation, we used a previously described approach of cytoplasmically loading thymocytes with OVA by hypotonic shock (Moore et al., 1988), followed by irradiation to promote apoptosis. Staining splenic cDC1 12 h after OVA-loaded AC transfer with an antibody specific for the H-2K^b^- SIINFEKL MHCI-peptide complex revealed a small population of positive cells (Fig. 2 B). Importantly, GPR34-deficiency led to a reduction in the frequency of H-2K^b^-SIINFEKL^+^ cDC1 (Fig. 2, B and C).

We next transferred OVA-loaded AC into hosts that harbored CellTrace Violet (CTV)-labeled OVA-specific OT-I T cells (Fig. 2 D). The sensitivity of this T cell activation assay allowed the use of two orders of magnitude fewer AC than for the efferocytosis assay. Flow cytometry analysis of the OT-I cells (Fig. S2 C) 1 day after OVA-loaded AC transfer revealed induction of the activation markers CD25, CD69, and PD-1 on a fraction of the cells in WT hosts (Fig. 2, E and F). The extent of OT-I T cell activation was markedly reduced in GPR34-deficient recipient mice (Fig. 2, E and F). OT-I T cell activation remained defective in GPR34-deficient mice at day 2 after OVA-loaded AC transfer (Fig. 2 G), a time point when little proliferation had occurred (Fig. S2 D).

### CD8 T cell proliferation is reduced when cDC1 lack GPR34

**Figure 3.**
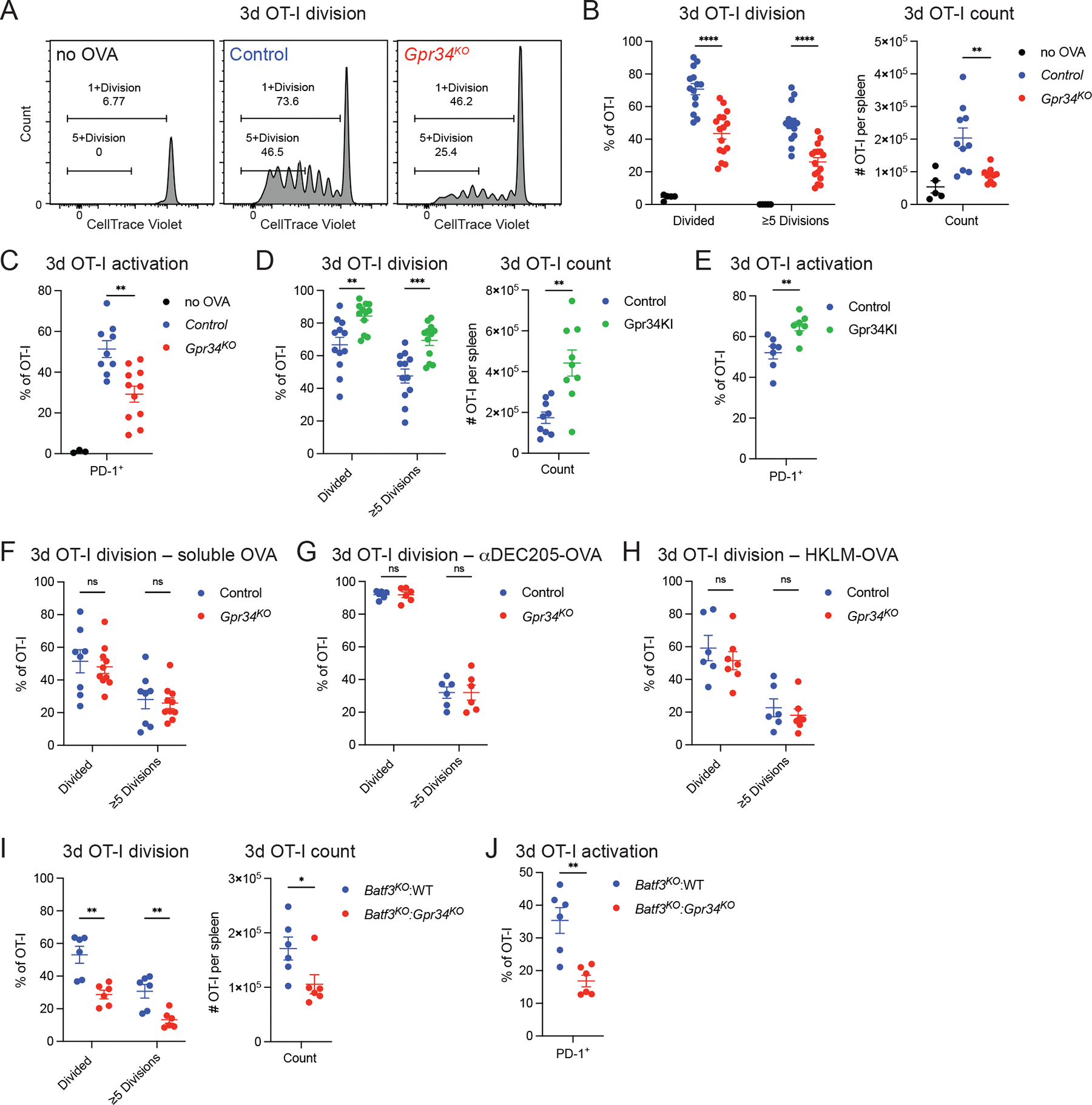
GPR34 in cDC1 enables cross-presentation of AC-associated antigen. **(A-J)** Splenic immune cells were analyzed by flow cytometry. **(A-C)** Analysis of OT-I response in mice unexposed to OVA or in control (WT or *Gpr34*^*Het*^) and *Gpr34*^*KO*^ mice 3 days after IV transfer of OVA-loaded thymocytes. **(A)** Representative flow cytometry histograms of OT-I cells, gated for the number of divisions based on CTV dilution and annotated with proliferation frequencies. **(B)** Left: Frequencies of OT-I division (no OVA, *n* = 5; control, *n* = 14; *Gpr34*^*KO*^, *n* = 16). Right: Number of OT-I cells per spleen (no OVA, *n* = 5; control, *n* = 10; *Gpr34*^*KO*^, *n* = 10). **(C)** Frequencies of PD-1^+^ OT-I cells (no OVA, *n* = 3; control, *n* = 9; *Gpr34*^*KO*^, *n* = 11). **(D and E)** Analysis of OT-I response in control (*Xcr1*^*Cre*^) and Gpr34KI (*Xcr1*^*Cre*^*R26*^*LSL-Gpr34Q340X-IRES-GFP*^) mice 3 days after IV transfer of OVA-loaded thymocytes. **(D)** Left: Frequencies of OT-I division (control, *n* = 12; Gpr34KI, *n* = 12). Right: Number of OT-I cells per spleen (control, *n* = 9; Gpr34KI, *n* = 9). **(E)** Frequencies of PD-1^+^ OT-I cells (control, *n* = 7; Gpr34KI, *n* = 7). **(F)** Frequencies of OT-I division in control (WT or *Gpr34*^*Het*^) and *Gpr34*^*KO*^ mice 3 days after IV injection of OVA (control, *n* = 8; *Gpr34*^*KO*^, *n* = 10). **(G)** Frequencies of OT-I division in control (WT or *Gpr34*^*Het*^) and *Gpr34*^*KO*^ mice 3 days after IV injection of αDEC205-OVA (control, *n* = 6; *Gpr34*^*KO*^, *n* = 6). **(H)** Frequencies of OT-I division in control (WT or *Gpr34*^*Het*^) and *Gpr34*^*KO*^ mice 3 days after IV transfer of HKLM-OVA (control, *n* = 6; *Gpr34*^*KO*^, *n* = 7). **(I and J)** Analysis of OT-I response 3 days after transfer of OVA-loaded thymocytes in mixed chimeric mice with a 50:50 mixture of *Batf3*^*KO*^ BM and of WT or *Gpr34*^*KO*^ BM reconstituted into WT hosts. **(I)** Left: Frequencies of OT-I division (*Batf3*^*KO*^:WT, *n* = 6; *Batf3*^*KO*^:*Gpr34*^*KO*^, *n* = 6). Right: Number of OT-I cells per spleen (*Batf3*^*KO*^:WT, *n* = 6; *Batf3*^*KO*^:*Gpr34*^*KO*^, *n* = 6). **(J)** Frequencies of PD-1^+^ OT-I cells (*Batf3*^*KO*^:WT, *n* = 6; *Batf3*^*KO*^:*Gpr34*^*KO*^, *n* = 6). In B-J, each data point represents an individual mouse, lines indicate means, and error bars represent the SEM. Data were pooled from two or more independent experiments. Statistical significance was determined by an unpaired *t* test corrected for multiple comparisons (Holm-Šídák). ns, not significant; *P < 0.05; **P < 0.01; ***P < 0.001; ****P < 0.0001.

Analysis at day 3 after OVA-loaded AC transfer revealed substantial OT-I CD8 T cell division in WT hosts (Fig. 3, A and B) as expected (Liu et al., 2002). The extent of OT-I T cell division was significantly reduced in GPR34-deficient mice (Fig. 3, A and B). OT-I activation at the day 3 time point, as assessed by PD-1 induction, was also compromised in GPR34-deficient hosts (Fig. 3 C). Staining for CD44 and CD62L revealed less efficient differentiation of OT-I cells to a CD44^hi^ and CD62L^lo^ state (Fig. S2, E and F). Performing similar analyses in mice expressing the GPR34 GOF allele in cDC1 showed that elevated GPR34 function was sufficient to support increased OT-I proliferation and activation marker expression in response to OVA-loaded AC (Fig. 3, D and E; and Fig. S2 G). In contrast, OT-I proliferation in response to soluble OVA (Fig. 3 F), to OVA targeted to the cDC1 surface molecule DEC205 (Fig. 3 G), or to HKLM-OVA (Fig. 3 H), was unaffected by GPR34 deficiency.

To confirm that GPR34 function in supporting AC-associated antigen cross-presentation reflected a role for the receptor in cDC1, we took advantage of BATF3-deficient mice that have a selective deficiency in cDC1 (Hildner et al., 2008). We first confirmed the role of BATF3-dependent cDC in supporting OT-I activation and proliferation in response to OVA-loaded AC by performing transfers to BATF3-deficient mice (Fig. S2, H-J). To test for a GPR34-intrinsic role within BATF3-dependent cDC1, we generated mixed chimeras using BATF3-deficient and GPR34-deficient BM donors, such that all the splenic cDC1 in the chimeras would be GPR34-deficient while cells of all other lineages would be 50% WT. Mixed chimeras made with BATF3-deficient and WT BM served as controls. OT-I cells were transferred into the chimeric mice prior to IV transfer of OVA-loaded AC. A similar defect in OT-I proliferation (Fig. 3 I), PD-1 induction (Fig. 3 J), and CD44 upregulation and CD62L downregulation (Fig. S2 K) was observed in the BATF3- and GPR34-deficient mixed chimeras as in GPR34-fully deficient hosts (Fig. 3, A-C). These findings indicate that cDC1 expression of GPR34 is necessary for an intact CD8 T cell proliferative response to AC-derived antigen.

### PLA1A is required for GPR34 function in cDC1 efferocytosis and cross-presentation

**Figure 4.**
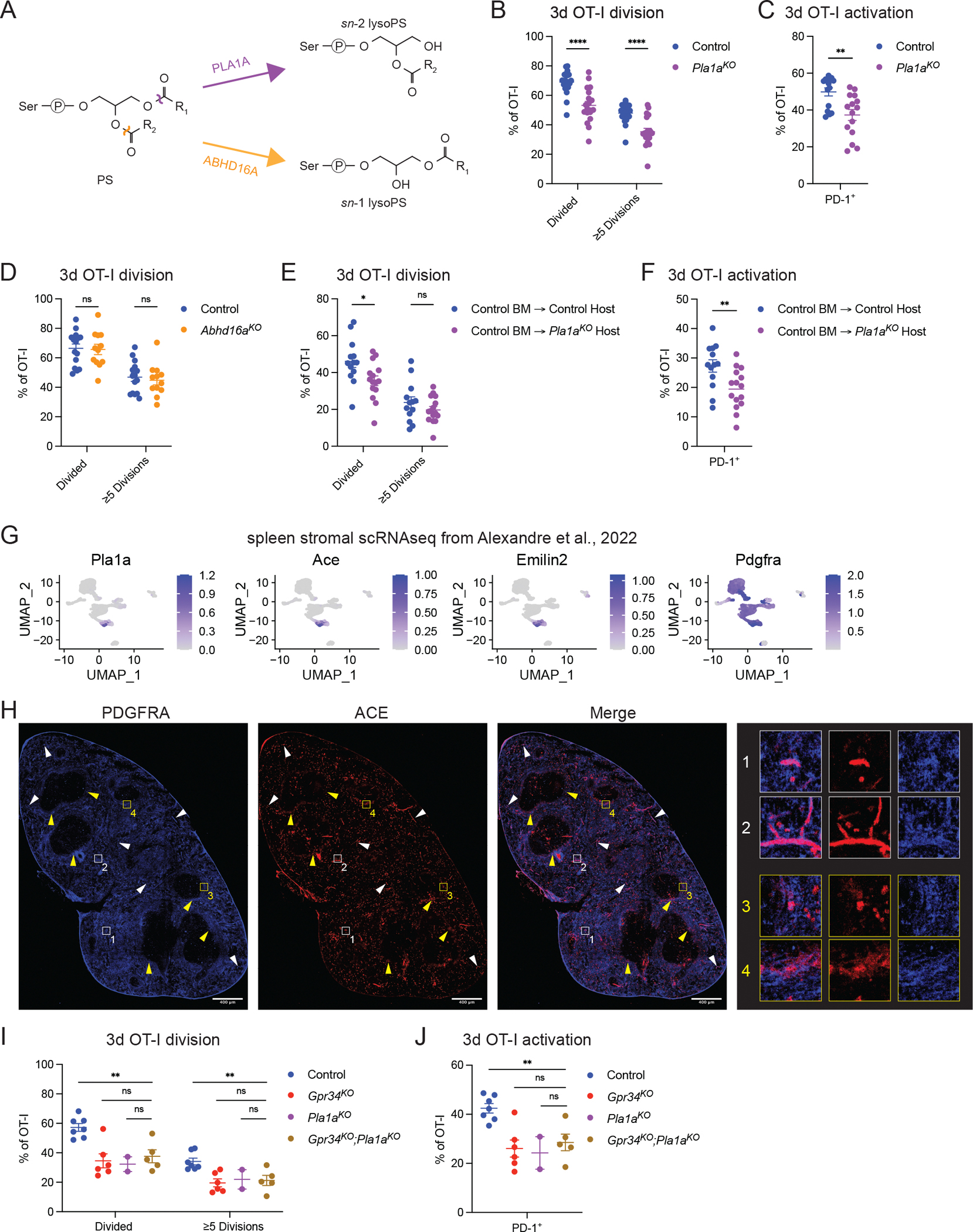
PLA1A acts in the GPR34 pathway to promote cDC1 cross-presentation. **(A)** Schematic of PS conversion to *sn*-2 and *sn*-1 lysoPS by PLA1A and ABHD16A, respectively. **(B-F, I, and J)** Splenic immune cells were analyzed by flow cytometry. **(B and C)** Analysis of OT-I response in control (WT or *Pa1a*^*Het*^) and *Pla1a*^*KO*^ mice 3 days after IV transfer of OVA-loaded thymocytes. **(B)** Frequencies of OT-I division (control, *n* = 21; *Pla1a*^*KO*^, *n* = 19). **(C)** Frequencies of PD-1^+^ OT-I cells (control, *n* = 15; *Pla1a*^*KO*^, *n* = 15). **(D)** Frequencies of OT-I division in control (*Abhd16a*^*Het*^) and *Abhd16a*^*KO*^ mice 3 days after IV transfer of OVA-loaded thymocytes (control, *n* = 15; *Abhd16a*^*KO*^, *n* = 12). **(E and F)** Analysis of OT-I response 3 days after transfer of OVA-loaded thymocytes in chimeric mice with control (WT or *Pa1a*^*Het*^) BM reconstituted into control (WT or *Pa1a*^*Het*^) or *Pla1a*^*KO*^ hosts. **(E)** Frequencies of OT-I division (control BM → control host, *n* = 13; control BM → *Pla1a*^*KO*^ host, *n* = 15). **(F)** Frequencies of PD-1^+^ OT-I cells (control BM → control host, *n* = 13; control BM → *Pla1a*^*KO*^ host, *n* = 15). **(G)** Seurat reanalysis of the mouse spleen stromal cell scRNAseq dataset from Alexandre et al. (2022). UMAPs show normalized expression of *Pla1a*, *Ace*, *Emilin2, and Pdgfra*. **(H)** Representative immunofluorescence image of a spleen section stained for PDGFRA and ACE from WT mice (*n* = 2). Scale bar, 400 μm. Numbered white and yellow boxes correspond to magnified views (right) containing examples of PDGFRA^+^ACE^+^ cells in the red pulp and MZ, respectively. White and yellow arrows indicate additional examples. **(I and J)** Analysis of OT-I response in control (WT or *Gpr34*^*Het*^;WT or *Pla1a*^*Het*^), *Gpr34*^*KO*^ (*Gpr34*^*KO*^;WT or *Pla1a*^*Het*^), *Pla1a*^*KO*^ (WT or *Gpr34*^*Het*^;*Pla1a*^*KO*^), and *Gpr34*^*KO*^;*Pla1a*^*KO*^ mice 3 days after IV transfer of OVA-loaded thymocytes. **(I)** Frequencies of OT-I division (control, *n* = 7; *Gpr34*^*KO*^, *n* = 6; *Pla1a*^*KO*^, *n* = 2; and *Gpr34*^*KO*^;*Pla1a*^*KO*^, *n* = 5). **(J)** Frequencies of PD-1^+^ OT-I cells (control, *n* = 7; *Gpr34*^*KO*^, *n* = 6; *Pla1a*^*KO*^, *n* = 2; and *Gpr34*^*KO*^;*Pla1a*^*KO*^, *n* = 5). In B-F, I, and J, each data point represents an individual mouse, lines indicate means, and error bars represent the SEM. Data were pooled from two or more independent experiments. Statistical significance was determined by an unpaired *t* test corrected for multiple comparisons (Holm-Šídák). ns, not significant; *P < 0.05; **P < 0.01; ****P < 0.0001.

The enzyme PLA1A catabolizes PS into *sn*-2 lysoPS, a potent GPR34 ligand (Kitamura et al., 2012) (Fig. 4 A). In mice lacking PLA1A, there were reduced proliferation of and PD-1 and CD44 induction on OT-I T cells in response to OVA-loaded AC (Fig. 4, B and C; and Fig. S2 L). ABHD16A has also been suggested to generate GPR34 ligands (Kamat et al., 2015; Yan et al., 2024) (Fig. 4 A), however, we did not observe any defect in OT-I proliferation in ABHD16A-deficient recipients (Fig. 4 D). In our previous work examining the effect of GPR34 GOF on the B cell response, we identified a role for PLA1A expression in omental stromal cells (Tam et al., 2024). To test the importance of stromal PLA1A in splenic cDC1 efferocytosis, we reconstituted PLA1A-deficient or control mice with control BM and used the chimeric mice as hosts for OT-I and OVA-loaded AC transfer. The day 3 OT-I proliferative response was reduced in the absence of PLA1A expression by radioresistant cells (Fig. 4 E), and there was less upregulation of PD-1 (Fig. 4 F). Analysis of a splenic stromal single-cell RNA sequencing (scRNAseq) data set (Alexandre et al., 2022) identified a small subset of *Pla1a*-expressing *Pdgfra*^+^ cells that had highly correlated expression of *Ace* and *Emilin2* (Fig. 4 G). In the absence of a verified anti-PLA1A antibody, we attempted to determine PLA1A distribution by taking advantage of a *lacZ* cassette present in the gene-targeted *Pla1a* locus. The liver highly expresses *Pla1a* mRNA (https://BioGPS.org) and β-galactosidase activity was readily detected in liver sections. However, the β-galactosidase activity in the spleen was below the detection limit of this assay (unpublished data). Given the tight correlation between *Pla1a* and *Ace* expression among stromal cells, we examined ACE distribution along with the broad stromal cell marker PDGFRA to provide insight into the distribution of cells likely competent to express PLA1A. ACE-expressing PGDFRA^+^ stromal cells were observed scattered in the red pulp and MZ (Fig. 4 H), a distribution that could allow close association between AC arriving via blood circulation and PLA1A-expressing cells. Additional ACE-expressing cells in the splenic red pulp that did not co-stain for PDGFRA may correspond to monocytes based on transcript expression data (https://www.immgen.org). We note that we were unable to test the impact of PLA1A deficiency on in vivo AC uptake using the mixed BM chimera approach employed in our GPR34 analysis due to PLA1A acting in a cDC1-extrinsic manner. Finally, to determine whether GPR34 and PLA1A function in the same pathway, we generated GPR34 and PLA1A double-deficient mice. The OT-I proliferative response and PD-1 upregulation in response to OVA-loaded AC was equally compromised in GPR34-deficient, PLA1A-deficient, and double-deficient mice (Fig. 4, I and J). These findings provide support for the conclusion that PLA1A functions to produce GPR34 ligands and thereby promote cDC1 efferocytosis.

## Concluding Remarks

Our findings establish that GPR34 acts intrinsically within cDC1 and in cooperation with PLA1A to support efferocytosis. We suggest a model where AC arriving via circulation in the splenic red pulp and MZ are engaged by stromal-derived PLA1A to generate *sn*-2 lysoPS that acts over short distances to promote cDC1 chemotaxis to and association with AC. Further engagement of GPR34 by AC membrane-associated lysoPS then cooperates with cDC1 PS receptors to mediate AC uptake. In accord with a model involving both chemotaxis and membrane-contact based signaling, in vitro studies have observed *sn*-2 lysoPS release from AC by PLA1A (Hosono et al., 2001) and provided evidence for direct exchange of lysoPS between producer and receiver cells (Uwamizu et al., 2025). GPR34 is a G_i_-coupled receptor, and G_i_-mediated induction of Rac activation and F-actin polymerization, which are broadly important for phagocytosis (Mylvaganam et al., 2021), likely contributes to AC uptake (Huang et al., 2014). Indeed, while Gi-coupled receptors are best known for their role in cell migration, an activity established for GPR34 (Kitamura et al., 2012; Tam et al., 2024; and this study), previous work has demonstrated that various G_i_-coupled receptors can promote particle phagocytosis (Cohen et al., 2022; Kamber et al., 2021; Kikuchi et al., 2005; Mai et al., 2015; Wen et al., 2019). GPR34 is highly expressed by microglia, and a study in zebrafish provided evidence that GPR34 contributes to microglia recruitment to a site of cell death (Lou et al., 2022). Microglia from GPR34-deficient mice showed reduced particle phagocytosis (Preissler et al., 2015) though not in all studies (Lin et al., 2024; Zhou et al., 2025). Our efforts to use in vitro approaches to assess the steps involved in lysoPS-mediated cDC1 migration and phagocytosis were unsuccessful (unpublished data) likely due to the rapid maturation events that occur in cDC upon isolation from tissues (Steinman et al., 2003) and maturation-induced downregulation of GPR34 (Jager et al., 2016). The high resolution (subcellular) intravital microscopy needed to more precisely determine the lysoPS–GPR34-dependent steps in splenic cDC1 efferocytosis awaits the development of next-generation deep tissue imaging techniques.

CD8 T cell proliferation in response to cDC1 that have taken up AC under non-inflammatory conditions is associated with tolerance (Ferguson et al., 2002; Liu et al., 2002; Steinman et al., 2000) while AC uptake under inflammatory conditions, such as in some tumor therapy settings or with bacterially infected AC, may contribute to immunity (Desch et al., 2011; Penteado et al., 2017; Torchinsky et al., 2009; Xie et al., 2022). It will be of interest in future work to test the impact of cDC1 GPR34 expression on CD8 T cell responses to tumors and to intracellular pathogens that induce apoptosis. A recent study found that GPR34 overexpression in natural killer cells could augment their infiltration of tumors (Kim et al., 2025, *Preprint*). Based on our findings, we suggest that lysoPS production from apoptotic tumor cells by stromal PLA1A may help recruit or retain cells engineered to overexpress GPR34.

Our findings show that GPR34 and PLA1A deficiency has an incomplete effect on cDC1 efferocytosis and induction of OT-I proliferation. This may reflect the action of other “find-me” and “eat-me” receptors on cDC1 (Arandjelovic and Ravichandran, 2015). Indeed, overlapping roles for multiple receptors may contribute to the general difficulty in establishing the in vivo mechanism of cDC1 efferocytosis (Belz et al., 2002; Nakayama et al., 2009; Perry et al., 2018; Schulz et al., 2002). Additionally, the recent finding of BATF3-dependent tumor cell-derived antigen cross-presentation by marginal metallophilic macrophages (Mauvais et al., 2025) raises the possibility of an additional cell type contributing to AC cross-presentation in the spleen. However, marginal metallophilic macrophages had low *Gpr34* transcript expression making it unlikely that the receptor contributes to their function (Fig. S3). The role of GPR34 expression in splenic cDC2 and F4/80^+^ macrophages is presently unclear, but the receptor might enhance their uptake of AC material under conditions not tested here, such as in the presence of PAMPs or in the absence of “don’t-eat-me” signals like CD47.

While our study provides evidence for a stromal contribution to the PLA1A needed for GPR34 efferocytosis in the spleen, the precise PLA1A-expressing stromal cell type(s) involved remain(s) to be defined. Moreover, although we favor a local expression model, our work does not exclude the possibility that systemic PLA1A contributes to local lysoPS production. In this regard, serum PLA1A has been identified as a biomarker of systemic lupus erythematosus disease activity (Iwata et al., 2021; Sawada et al., 2019). Elevated systemic PLA1A may influence how AC are taken up in the disease setting. Previous mouse studies have implicated ABHD16A in GPR34 ligand generation (Lin et al., 2024; Yan et al., 2024; Zhou et al., 2025). More work will be needed to determine whether both PLA1A and ABHD16A contribute to GPR34 ligand production in some settings. However, given that the form of lysoPS generated by PLA1A is a markedly more potent GPR34 ligand than the form generated by ABHD16A, we favor the view that PLA1A will be more broadly important for GPR34 function.

## Materials and methods

### Mice

*Gpr34*^*KO*^ (Liebscher et al., 2011), *Xcr1*^*Cre*^ (Ferris et al., 2020), *R26*^*LSL-Gpr34Q340X-IRES-GFP*^ (Tam et al., 2024), OT-I (Hogquist et al., 1994), and *Batf3*^*KO*^ (Hildner et al., 2008) mice have been previously described. *Pla1a*^*KO*^ mice (Tam et al., 2024) harbor a *Pla1a* locus that was disrupted by gene trap insertion of a *lacZ* cassette (Skarnes et al., 2011). *Abhd16a*^*KO*^ mice were generated from embryonic stem cells of a C56BL/6N background purchased from EUCOMM as previously described (Kamat et al., 2015). Mice were used at 7–16 wk of age. Congenic hosts for BM chimeras and recipients for transfer experiments were either B6 CD45.1 (002014) mice bred internally from founders ordered from The Jackson Laboratory or B6-Ly5.1/Cr (564) mice purchased from the National Cancer Institute at Charles River at age 6–8 wk. To produce BM chimeric mice, hosts were lethally irradiated with 900 cGy x-ray irradiation (split dose separated by 3 h), followed by IV injection of BM cells (typically 5 M total) from donors. Chimeras were used at 7–24 wk after reconstitution. Mice were co-caged with littermates for all experiments. All data are representative of male and female mice. Control and experimental treatments were administered to age- and sex-matched mice that had been allocated to groups randomly, with sample sizes chosen based on previous experience. The investigators were not blinded. Animals were housed in a specific pathogen–free environment in the Laboratory Animal Research Center at UCSF, and all experiments conformed to ethical principles and guidelines approved by the UCSF Institutional Animal Care and Use Committee.

### Cell preparation

For lymphocyte analysis, cell suspensions were prepared from spleen by gentle mashing in MACS buffer (PBS [Thermo Fisher Scientific] containing 2% newborn calf serum [NBCS] [Thermo Fisher Scientific] and 1 mM EDTA [Thermo Fisher Scientific]) through a 70-μm strainer. For myeloid cell analysis, the spleen was dissected and minced using scissors. The minced spleen tissue was incubated and gently rotated for 30 min at 37°C in 1 ml of RPMI (Thermo Fisher Scientific) with 2% NBCS, 10 mM HEPES (Thermo Fisher Scientific), 1 mg/ml collagenase type IV (Worthington), and 20 μg/ml DNase I (Sigma-Aldrich). To stop the digestion, 20 μL of 500 mM EDTA was added to each sample, and the sample was incubated on ice for 10 min. The digested spleen tissue was mashed through a 100-μm strainer and washed with MACS buffer.

### Flow cytometry

Cells were blocked with 2.4G2 antibody (Bio X Cell) and stained for 30 min on ice in MACS buffer. The following antibodies were used: CD11b-BUV496 (Thermo Fisher Scientific), CD19-BUV563 (Thermo Fisher Scientific), CD4-BUV615 (Thermo Fisher Scientific), TCRb-BUV737 (Thermo Fisher Scientific), I-A/I-E-BUV805 (Thermo Fisher Scientific), CD45.2-BV421 (BioLegend), TCRb-BV421 (BioLegend), CD8a-PB (BioLegend), CD19-PB (BioLegend), CD8a-BV510 (BioLegend), CD62L-BV510 (BioLegend), CD11b-BV570 (BioLegend), CD25-BV605 (BioLegend), CD86-BV605 (BioLegend), Ly6C-BV650 (BioLegend), Va2-BV650 (Thermo Fisher Scientific), CD4-BV711 (Thermo Fisher Scientific), CD69-BV711 (BioLegend), SA-BV711 (Thermo Fisher Scientific), CD11b-BV785 (BioLegend), CD44-BV785 (BioLegend), CD45.1-BV785 (BioLegend), CD11c-FITC (BioLegend), CD45.1-FITC (BioLegend), F4/80-FITC (BioLegend), I-Ab-FITC (Thermo Fisher Scientific), XCR1-FITC (BioLegend), F4/80-PerCP (BioLegend), B220-PerCP/Cy5.5 (Cytek), CD8a-PerCP/Cy5.5 (Cytek), I-Ab-PerCP/Cy5.5 (BioLegend), TCRb-PerCP/Cy5.5 (Cytek), SIRPa-PerCP-eFluor 710 (Thermo Fisher Scientific), CD11b-PE (BioLegend), CD11c-PE (BioLegend), CD45.1-PE (BioLegend), F4/80-PE (BioLegend), SA-PE (Thermo Fisher Scientific), Thy1.1-PE (BioLegend), Va2-PE (BioLegend), XCR1-PE (BioLegend), SIRPa-PE/Dazzle 594 (BioLegend), CD11c-PE/Cy7 (Cytek), CD69-PE/Cy7 (BioLegend), PD-1-PE/Cy7 (BioLegend), CD45.1-APC (Cytek), CD62L-APC (BioLegend), F4/80-APC (Cytek), XCR1-AF647 (BioLegend), B220-AF700 (BioLegend), CD45.1-AF700 (BioLegend), CD45.2-AF700 (BioLegend), B220-APC/Fire 810 (BioLegend), F4/80-biotin (BioLegend), XCR1-biotin (BioLegend). Dead cells were excluded using Fixable Viability Dye eFluor 780 (eBioscience).

For CCR7 surface staining, cells were stained with CCR7-biotin (BioLegend) for 45 min on ice, followed by SA-BV711 (Thermo Fisher Scientific) and other surface markers for 30 min on ice. For H-2K^b^-SIINFEKL MHCI-peptide complex surface staining, cells were stained with the APC-conjugated 25-D1.16 antibody (BioLegend) for 45 min on ice.

GPR34 surface staining was performed as previously described (Tam et al., 2024). Briefly, a polyclonal rabbit anti-GPR34 antibody (custom generated at Biomatik) was cross absorbed on a *Gpr34*^*KO*^ spleen cell suspension overnight to reduce non-specific binding. After blocking, WT and *Gpr34*^*KO*^ cells were stained with the polyclonal reagent for 1 h at RT, followed by donkey anti-rabbit biotin-SP (Jackson ImmunoResearch) for 20 min on ice, and finally SA-AF647 (Invitrogen) and other surface markers for 30 min on ice.

All samples were run on a Cytek Aurora. Flow cytometry data were analyzed using FlowJo (v10.10.0).

### Uptake experiments

For efferocytosis experiments, AC were prepared by exposing a cell suspension of WT thymocytes and splenocytes to 1,350 cGy x-ray irradiation, followed by dye labeling with CellTracker DR (Invitrogen) according to the manufacturer’s instructions. 20 million DR-labeled AC were IV injected via the retroorbital route. At 1 h or 12 h after injection, mice were euthanized, and uptake of DR-labeled AC in the spleen was assessed by flow cytometry.

For assessment of soluble protein uptake, 50 μg of OVA-AF647 (Thermo Fisher Scientific) was IV injected 1 h before analysis.

For assessment of bacterial uptake, a saturated overnight culture of *L. monocytogenes* was diluted in Brain Heart Infusion media (Thermo Fisher Scientific) and grown to an OD of 0.5 corresponding to a concentration of 6.7 × 10^8^ *Listeria* per ml. The bacteria were dye-labeled with DR and subsequently heat-killed for 1 h at 80ºC (Kretzer et al., 2016). 3 × 10^9^ DR-labeled HKLM were IV injected 1 h before analysis.

For assessment of eryptotic RBC uptake, eryptotic RBCs were prepared as previously described (Larsson et al., 2016). Briefly, 0.5ml of blood was collected via cardiac puncture of a WT mouse into a MicroTainer EDTA tube (Thermo Fisher Scientific). RBCs were labeled using the PKH26 Red Fluorescent Cell Linker Kit (Sigma-Aldrich) according to manufacturer’s instructions. Labeled RBCs were stimulated for 1 h with 1 μM ionomycin (Sigma-Aldrich) at a concentration of 1 × 10^8^ RBCs per ml in RPMI with 10 mM HEPES. After washing, 1 × 10^8^ PKH26-labeled eryptotic RBCs were IV injected 1 h before analysis.

### OT-I response experiments

Spleen and mesenteric, inguinal, brachial, axillary, and cervical lymph nodes from congenic OT-I mice were gently mashed in RPMI containing 10% FBS (Omega Scientific), 10 mM HEPES, 2 mM glutamine, 55 μM 2-mercaptoethanol, and 50 U penicillin/streptomycin (Thermo Fisher Scientific) through a 70-μm strainer. The cell suspension was negatively selected for CD8 T cells using magnetic EasySep Streptavidin RapidSpheres (StemCell Technologies) and a cocktail of biotin-conjugated antibodies against B220 (Cytek), CD4 (BioLegend), CD11c (BioLegend), CD19 (BioLegend), CD44 (BioLegend), CD49b (BioLegend), F4/80 (BioLegend), TCRgd (Thermo Fisher Scientific), and TER-119 (BioLegend). Isolated CD8 T cells were dye-labeled with CTV. 1 million CTV^+^CD8a^+^TCRb^+^Va2^+^ OT-I cells were IV transferred to recipient mice 1 day before exposure to OVA.

For assessment of OVA peptide presentation on MHC class I, osmotically loaded thymocytes were prepared as previously described (Moore et al., 1988). Briefly, thymocytes from WT mice were gently mashed in PBS. 500 million thymocytes were incubated for 10 min at 37°C in 10 ml of hypertonic medium containing 0.5 M sucrose (Sigma-Aldrich), 10% wt/vol polyethylene glycol 1,000 (Mallinckrodt Baker), 10 mM HEPES, and 10 mg/ml OVA (Sigma-Aldrich) in RPMI. After the addition of 130 ml of prewarmed hypotonic medium (40% H_2_O, 60% RPMI) for 2 min at 37°C, cells were immediately spun, washed with PBS, and subjected to 1,350 cGy of x-ray irradiation. 40 million OVA-loaded thymocytes were IV transferred to recipient mice. 12 h later, cell surface H-2K^b^-SIINFEKL MHCI-peptide complexes were assessed by flow cytometry.

For assessment of OT-I response to AC-associated OVA, osmotically OVA-loaded thymocytes were prepared similar to above, except that WT thymocytes were gently mashed in RPMI with 10 mM HEPES. 10 million thymocytes were incubated for 10 min at 37°C in 1 ml of hypertonic medium followed by the addition of 13 ml of prewarmed hypotonic medium, washing, and x-ray irradiation. 50,000 OVA-loaded thymocytes were IV transferred to recipient mice. *Batf3*^*KO*^ mixed chimeric mice received 100,000 OVA-loaded thymocytes to compensate for having half the abundance of cDC1.

For assessment of OT-I response to soluble OVA or to OVA targeted to cDC1, 3 μg OVA or 30 ng αDEC205-OVA (Boscardin et al., 2006) was IV injected, respectively. For assessment of OT-I response to bacteria-associated OVA, 3 × 10^9^ HKLM-OVA were IV injected.

At 1, 2, or 3 days after injection of various forms of OVA, mice were euthanized, and OT-I activation, proliferation, and differentiation in the spleen were assessed by flow cytometry.

### Transwell migration assay

Spleens were dissected and minced using scissors. The minced spleen tissue was then digested in DMEM (Thermo Fisher Scientific) containing 2% FBS, 10 mM HEPES, 1 mg/ml collagenase type IV, 20 ug/ml DNase I, and 50 U penicillin/streptomycin for 10 min at 37°C and mashed through a 70-μm strainer, followed by RBC lysis with ACK lysing buffer (Thermo Fisher Scientific). Cell suspensions were washed once in prewarmed migration media (RPMI containing 0.5% fatty acid-free BSA, 10 mM HEPES, and 50 U penicillin/streptomycin) and resuspended at 2 × 10^7^ cells per ml and resensitized for 25 min in a 37°C water bath. 100 μl of cells (2 × 10^6^ cells) was added on top of transwells (5-μm pore; Corning) with CCL19 (R&D Systems) or lysoPS 18:1 (Avanti Polar Lipids) in migration media (600 μl) in the bottom chamber. The cells were allowed to migrate for 3 h, after which the cells in the bottom well were analyzed and counted by flow cytometry.

### Published scRNAseq and bulk RNAseq data analysis

A spleen stromal cell scRNAseq dataset (Alexandre et al., 2022) and a mononuclear phagocyte scRNAseq dataset (Tadepalli et al., 2025) were reanalyzed using the Seurat R package (https://satijalab.org/seurat/). To find a surrogate marker for *Pla1a*^*+*^ stromal cells, dropout gene expression was imputed using Rmagic (van Dijk et al., 2018), and the Pearson correlation between *Pla1a* and every other gene was calculated. *Ace* and *Emilin2* were the two highest correlated genes with coefficients of 0.96 and 0.90, respectively.

Bulk RNAseq data from splenic marginal metallophilic macrophages (Mauvais et al., 2025) were processed using the same pipeline as in Brown et al. (2019). The results were then analyzed alongside splenic cDC1 bulk RNAseq data (Brown et al., 2019) using DESeq2 (Love et al., 2014) and edgeR (Robinson et al., 2010).

### Immunofluorescence staining and microscopy

Spleen was fresh-frozen in OCT. 10-μm cryosections were fixed by acetone (Thermo Fisher Scientific) for 10 min, dried for 1 h, and stained with PDGFRA-PE (Thermo Fisher Scientific), XCR1-PE (BioLegend), CD169-AF647 (BioLegend), goat anti-ACE (Thermo Fisher Scientific) (Cao et al., 2023), and donkey anti-goat IgG (H+L) AF647 (Thermo Fisher Scientific). Primary antibody incubation was performed for 2 h at room temperature, followed by secondary staining for 2 h at room temperature. Images were acquired with a HP PL FLUOTAR 10X/0.2 AIR objective on a Stellaris DIVE confocal microscope (Leica) using LAS X software (Leica) and subsequently processed using ImageJ (v2.16.0).

### Statistical analyses

Data were analyzed using unpaired or paired *t* tests corrected for multiple comparisons (Holm-Šídák) as specified in the figure legends. Prism software (GraphPad v10.4.2) was used for all statistical analyses and to generate plots. Each experiment was repeated at least two times, unless otherwise indicated in the figure legends. In summary graphs, points indicate individual samples, horizontal lines are means, and error bars represent the SEM. Levels of significance were defined as *P < 0.05, **P < 0.01, ***P < 0.001, and ****P < 0.0001.

## Supplementary Material

Figure S1 Figure S1. Characterization of GPR34-deficient cDC1.
**(A, C, and D)** Splenic immune cells were analyzed by flow cytometry.
**(A)** Representative flow cytometry plots showing gating strategy for cDC1, cDC2, F4/80^+^ MΦ, and CCR7 expression on cDC1.
**(B)** Seurat reanalysis of the mouse single cell RNA sequencing dataset from Tadepalli et al. (2025). Dot plot shows the average expression of and percent of cells expressing the indicated genes within the indicated cell subsets.
**(C)** Frequencies of DC populations in control (WT or *Gpr34*^*Het*^) and *Gpr34*^*KO*^ mice (control, *n* = 8; *Gpr34*^*KO*^, *n* = 8).
**(D)** Competitive competencies of DC population frequencies in mixed chimeric mice made by reconstituting WT hosts with a 50:50 mixture of control WT BM and of test WT or *Gpr34*^*KO*^ BM (WT:WT, *n* = 13; WT:*Gpr34*^*KO*^, *n* = 12).
**(E)** Representative immunofluorescence images of spleen sections stained for CD169 and XCR1 from control (WT or *Gpr34*^*Het*^) (*n* = 4) and *Gpr34*^*KO*^ mice (*n* = 4). CD169 highlights the inner marginal zone (MZ). RP, red pulp; WP, white pulp. Scale bar, 400 μm.
**(F)** Spleen cells were prepared from control (*Gpr34*^*Het*^), *Gpr34*^*KO*^, and Gpr34KI mice and assayed for transwell migration to the indicated concentrations of CCL19 or LysoPS. Bar graph shows the frequency of cDC1 migration relative to input (nil, *n* = 4; CCL19, *n* = 4; lysoPS 18:1 200nM, *n* = 4; lysoPS 18:1 1μM, *n* = 4; lysoPS 18:1 5μM, *n* = 4).
In C and D, each data point represents an individual mouse, lines indicate means, and error bars represent the SEM. In F, solid bars indicate means and error bars represent the SD. Data were pooled from two or more independent experiments. In C, D, and F, statistical significance was determined by an unpaired *t* test corrected for multiple comparisons (Holm-Šídák).
ns, not significant; ****P < 0.0001.

Figure S2 Figure S2. GPR34 and PLA1A support the activation and differentiation of AC-associated antigen-specific CD8 T cells.
**(A-L)** Splenic immune cells were analyzed by flow cytometry.
**(A)** Mixed chimeric mice were analyzed 12 h after IV transfer of DR-labeled AC. cDC1 were classified into two subsets: AC^−^ if DR^−^ and AC^+^ if DR^+^. Left: Frequency of CCR7^+^ cDC1 for the indicated subsets (WT, *n* = 9; *Gpr34*^*KO*^, *n* = 10). Right: Competitive competencies of CCR7^+^ cDC1 frequency for the indicated subsets (WT:WT, *n* = 9; WT:*Gpr34*^*KO*^, *n* = 10).
**(B)** Mixed chimeric mice were analyzed 12 h after IV transfer of DR-labeled or OVA-loaded AC. cDC1 were classified into two subsets: AC^−^ if DR^−^ or H-2K^b^- SIINFEKL^−^ and AC^+^ if DR^+^ or H-2K^b^- SIINFEKL^+^. Left: gMFI of CD86 on cDC1 for the indicated subsets (WT, *n* = 8; *Gpr34*^*KO*^, *n* = 10). Right: Competitive competencies of CD86 gMFI on cDC1 for the indicated subsets (WT:WT, *n* = 8; WT:*Gpr34*^*KO*^, *n* = 10).
**(C)** Representative flow cytometry plots showing gating strategy for OT-I cells.
**(D)** Frequencies of OT-I division in control (WT or *Gpr34*^*Het*^) and *Gpr34*^*KO*^ mice 2 days after transfer of OVA-loaded thymocytes (control, *n* = 10; *Gpr34*^*KO*^, *n* = 10).
**(E and F)** Analysis of OT-I response in mice unexposed to OVA or in control (WT or *Gpr34*^*Het*^) and *Gpr34*^*KO*^ mice 3 days after IV transfer of OVA-loaded thymocytes.
**(E)** Representative flow cytometry histograms of OT-I cells, gated for CD62L^+^CD44^–^, CD62L^+^CD44^+^, and CD62L^–^CD44^+^ subsets and annotated with the respective frequencies.
**(F)** Frequencies of CD62L^+^CD44^–^, CD62L^+^CD44^+^, and CD62L^–^CD44^+^ OT-I cells (no OVA, *n* = 5; control, *n* = 14; *Gpr34*^*KO*^, *n* = 16).
**(G)** Frequencies of CD62L^+^CD44^–^, CD62L^+^CD44^+^, and CD62L^–^CD44^+^ OT-I cells in control (*Xcr1*^*Cre*^) and Gpr34KI (*Xcr1*^*Cre*^*R26*^*LSL-Gpr34Q340X-IRES-GFP*^) mice 3 days after IV transfer of OVA-loaded thymocytes.
**(H-J)** Analysis of OT-I response in control (*Batf3*^*Het*^) and *Batf3*^*KO*^ mice 3 days after IV transfer of OVA-loaded thymocytes.
**(H)** Frequencies of OT-I division (control, *n* = 6; *Batf3*^*KO*^, *n* = 5).
**(I)** Frequencies of PD-1^+^ OT-I cells (control, *n* = 6; *Batf3*^*KO*^, *n* = 5).
**(J)** Frequencies of CD62L^+^CD44^–^, CD62L^+^CD44^+^, and CD62L^–^CD44^+^ OT-I cells (control, *n* = 6; *Batf3*^*KO*^, *n* = 5).
**(K)** Frequencies of CD62L^+^CD44^–^, CD62L^+^CD44^+^, and CD62L^–^CD44^+^ OT-I cells 3 days after IV transfer of OVA-loaded thymocytes in mixed chimeric mice that had been made by reconstituting WT hosts with a 50:50 mixture of *Batf3*^*KO*^ BM and of WT or *Gpr34*^*KO*^ BM (*Batf3*^*KO*^:WT, *n* = 6; *Batf3*^*KO*^: *Gpr34*^*KO*^, *n* = 6).
**(L)** Frequencies of CD62L^+^CD44^–^, CD62L^+^CD44^+^, and CD62L^–^CD44^+^ OT-I cells in control (WT or *Pa1a*^*Het*^) and *Pla1a*^*KO*^ mice 3 days after IV transfer of OVA-loaded thymocytes (control, *n* = 21; *Pla1a*^*KO*^, *n* = 19).
In A, B, D, and F-L, each data point represents an individual mouse, lines indicate means, and error bars represent the SEM. Data were pooled from two or more independent experiments. Statistical significance was determined by an unpaired *t* test corrected for multiple comparisons (Holm-Šídák).
ns, not significant; *P < 0.05; **P < 0.01; ***P < 0.001; ****P < 0.0001. gMFI, geometric mean fluorescence intensity.

Figure S3 Figure S3. Splenic marginal metallophilic macrophages minimally express GPR34.
**(A)** Reanalysis of bulk RNA-sequencing datasets for splenic marginal metallophilic macrophages (MMM) (Mauvais et al., 2025) and cDC1 (Brown et al., 2019). Heatmap comparing normalized expression of *Gpr34* and selected myeloid-associated genes.

Online supplemental material

Fig. S1 characterizes GPR34-deficient cDC1. Fig. S2 shows supporting data for the role of GPR34 and PLA1A in the cross-presentation of AC-associated antigen. Fig. S3 compares the gene expression profiles of cDC1 and marginal metallophilic macrophages.

## Data Availability

The reanalyzed scRNAseq datasets in Fig. 4 G (Alexandre et al., 2022) and in Fig. S1 B (Tadepalli et al., 2025) are openly available in GEO (accessions GSE167002 and GSE24350, respectively). The reanalyzed bulk RNAseq datasets for marginal metallophilic macrophages (Mauvais et al., 2025) and cDC1 (Brown et al., 2019) in Fig. S3 A are openly available at EBI (S-BSST950) and GEO (GSE130201), respectively. All other data are available in the article itself and its supplementary materials and are also available upon request from the corresponding author.
