# Supplementary figures and images for: Splenic cDC1 efferocytosis and cross-presentation to CD8 T cells are promoted by GPR34 and lysophosphatidylserine
Source: J Exp Med. Author manuscript; Available in PMC 2025 Dec 20. (PMC12717802; doi:10.1084/jem.20250887)

A

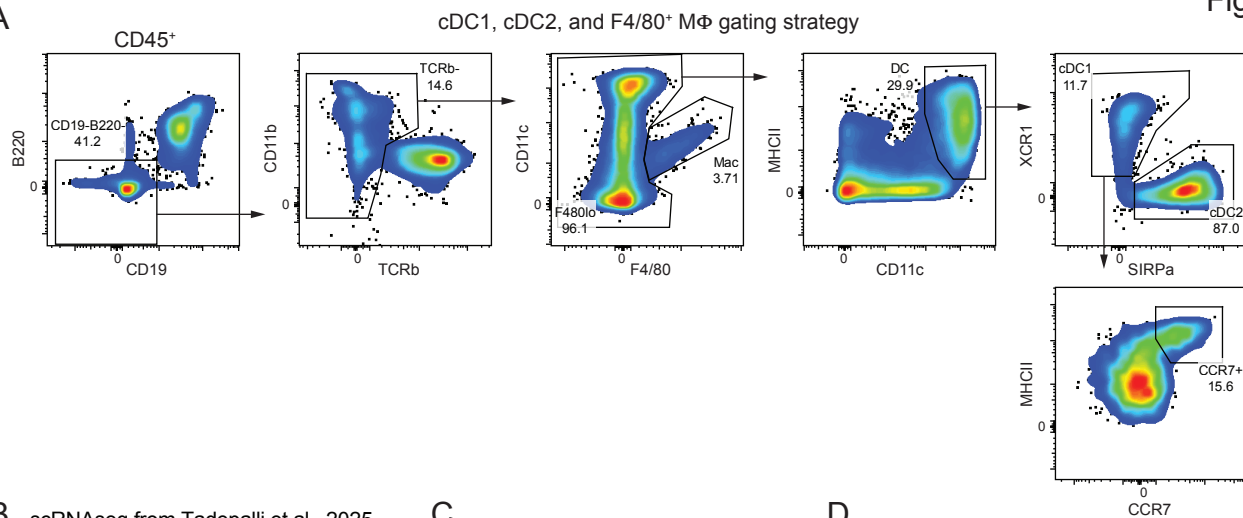

B

scRNAseq from Tadeapalli et al., 2025

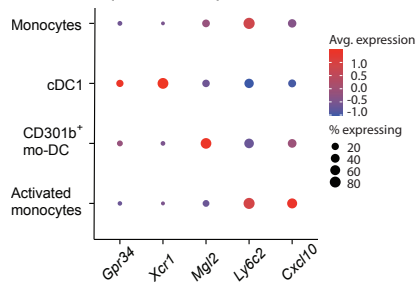

C

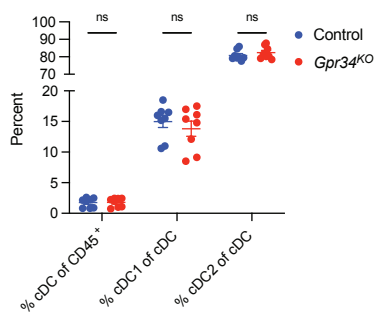

D

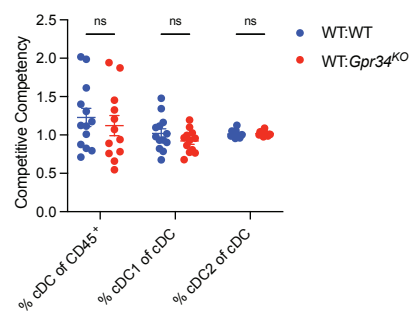

E

Control

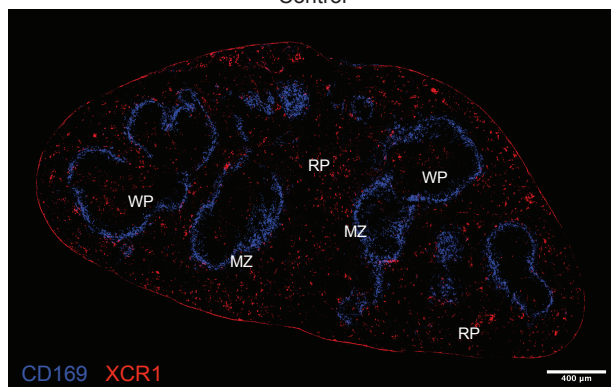*Gpr34*<sup>KO</sup>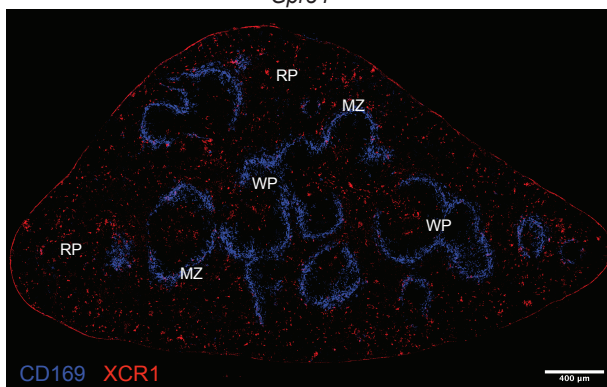

F

Spleen cDC1 Transwell Migration

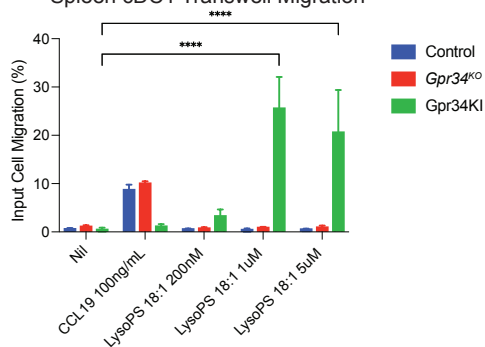

Supplement: Figure S1 — Characterization of GPR34-deficient cDC1. (A, C, and D) Splenic immune cells were analyzed by flow cytometry. (A) Representative flow cytometry plots showing gating strategy for cDC1, cDC2, F4/80+ MΦ, and CCR7 expression on cDC1. (B) Seurat reanalysis of the mouse single cell RNA sequencing dataset from Tadepalli et al. (2025). Dot plot shows the average expression of and percent of cells expressing the indicated genes within the indicated cell subsets. (C) Frequencies of DC populations in control (WT or Gpr34Het) and Gpr34KO mice (control, n = 8; Gpr34KO, n = 8). (D) Competitive competencies of DC population frequencies in mixed chimeric mice made by reconstituting WT hosts with a 50:50 mixture of control WT BM and of test WT or Gpr34KO BM (WT:WT, n = 13; WT:Gpr34KO, n = 12). (E) Representative immunofluorescence images of spleen sections stained for CD169 and XCR1 from control (WT or Gpr34Het) (n = 4) and Gpr34KO mice (n = 4). CD169 highlights the inner marginal zone (MZ). RP, red pulp; WP, white pulp. Scale bar, 400 μm. (F) Spleen cells were prepared from control (Gpr34Het), Gpr34KO, and Gpr34KI mice and assayed for transwell migration to the indicated concentrations of CCL19 or LysoPS. Bar graph shows the frequency of cDC1 migration relative to input (nil, n = 4; CCL19, n = 4; lysoPS 18:1 200nM, n = 4; lysoPS 18:1 1μM, n = 4; lysoPS 18:1 5μM, n = 4). In C and D, each data point represents an individual mouse, lines indicate means, and error bars represent the SEM. In F, solid bars indicate means and error bars represent the SD. Data were pooled from two or more independent experiments. In C, D, and F, statistical significance was determined by an unpaired t test corrected for multiple comparisons (Holm-Šídák). ns, not significant; ****P < 0.0001. [file NIHMS2122604-supplement-Figure_S1.pdf]

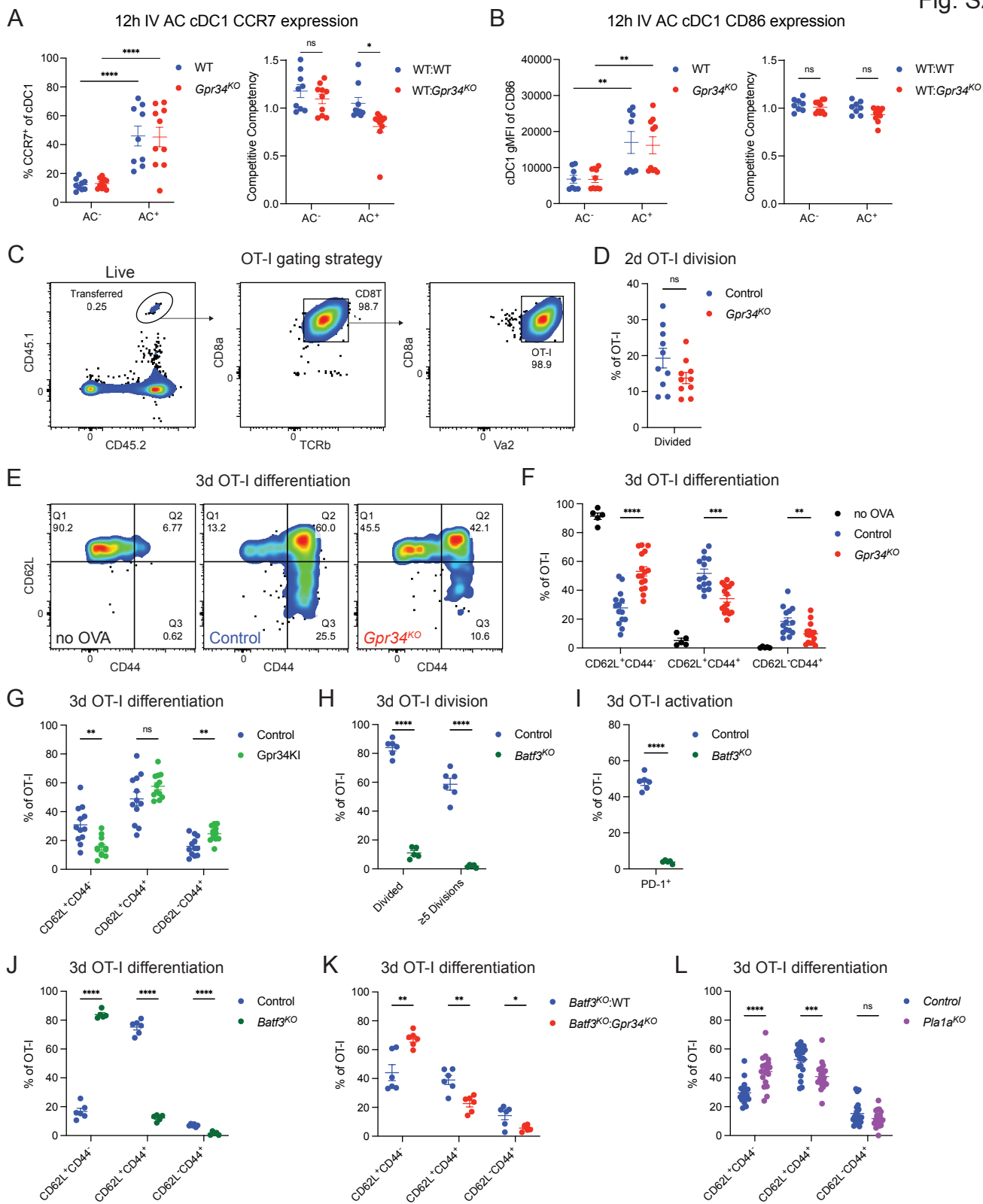

Supplement: Figure S2 — GPR34 and PLA1A support the activation and differentiation of AC-associated antigen-specific CD8 T cells. (A-L) Splenic immune cells were analyzed by flow cytometry. (A) Mixed chimeric mice were analyzed 12 h after IV transfer of DR-labeled AC. cDC1 were classified into two subsets: AC− if DR− and AC+ if DR+. Left: Frequency of CCR7+ cDC1 for the indicated subsets (WT, n = 9; Gpr34KO, n = 10). Right: Competitive competencies of CCR7+ cDC1 frequency for the indicated subsets (WT:WT, n = 9; WT:Gpr34KO, n = 10). (B) Mixed chimeric mice were analyzed 12 h after IV transfer of DR-labeled or OVA-loaded AC. cDC1 were classified into two subsets: AC− if DR− or H-2Kb- SIINFEKL− and AC+ if DR+ or H-2Kb- SIINFEKL+. Left: gMFI of CD86 on cDC1 for the indicated subsets (WT, n = 8; Gpr34KO, n = 10). Right: Competitive competencies of CD86 gMFI on cDC1 for the indicated subsets (WT:WT, n = 8; WT:Gpr34KO, n = 10). (C) Representative flow cytometry plots showing gating strategy for OT-I cells. (D) Frequencies of OT-I division in control (WT or Gpr34Het) and Gpr34KO mice 2 days after transfer of OVA-loaded thymocytes (control, n = 10; Gpr34KO, n = 10). (E and F) Analysis of OT-I response in mice unexposed to OVA or in control (WT or Gpr34Het) and Gpr34KO mice 3 days after IV transfer of OVA-loaded thymocytes. (E) Representative flow cytometry histograms of OT-I cells, gated for CD62L+CD44–, CD62L+CD44+, and CD62L–CD44+ subsets and annotated with the respective frequencies. (F) Frequencies of CD62L+CD44–, CD62L+CD44+, and CD62L–CD44+ OT-I cells (no OVA, n = 5; control, n = 14; Gpr34KO, n = 16). (G) Frequencies of CD62L+CD44–, CD62L+CD44+, and CD62L–CD44+ OT-I cells in control (Xcr1Cre) and Gpr34KI (Xcr1CreR26LSL-Gpr34Q340X-IRES-GFP) mice 3 days after IV transfer of OVA-loaded thymocytes. (H-J) Analysis of OT-I response in control (Batf3Het) and Batf3KO mice 3 days after IV transfer of OVA-loaded thymocytes. (H) Frequencies of OT-I division (control, n = 6; Batf3KO, n = 5). [file NIHMS2122604-supplement-Figure_S2.pdf]

## A RNAseq from Mauvais et al., 2025 and Brown et al., 2019

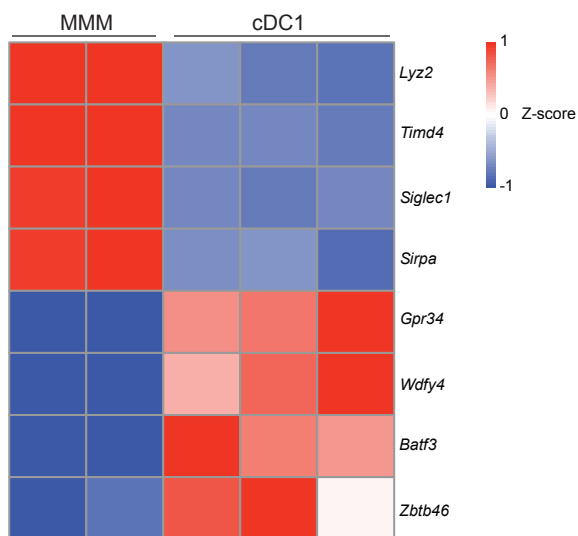

Supplement: Figure S3 — Splenic marginal metallophilic macrophages minimally express GPR34. (A) Reanalysis of bulk RNA-sequencing datasets for splenic marginal metallophilic macrophages (MMM) (Mauvais et al., 2025) and cDC1 (Brown et al., 2019). Heatmap comparing normalized expression of Gpr34 and selected myeloid-associated genes. [file NIHMS2122604-supplement-Figure_S3.pdf]
